# Supplementary material for: Structure and elasticity of CaC2O5 suggests carbonate contribution to the seismic anomalies of Earth’s mantle
Source: Nat Commun. 2024 Jan 25;15:755. doi: 10.1038/s41467-024-44925-9 (PMC10811330; doi:10.1038/s41467-024-44925-9)
Supplement: Supplementary file 1 — Supplementary Information [file 41467_2024_44925_MOESM1_ESM.pdf]

Supplementary Information

Structure and elasticity of CaC_2O_5 suggests carbonate contribution to the seismic anomalies of Earth's mantle

Hanyu Wang[1,2], Lei Liu[2*], Zihan Gao[2], Longxing Yang[1,2], Gerile Naren[2], Shide Mao[1*]

[1]State Key Laboratory of Geological Processes and Mineral Resources, and School of Earth Sciences and Resources, China University of Geosciences, 100083, Beijing, China.

[2]United Laboratory of High-Pressure Physics and Earthquake Science, Institute of Earthquake Forecasting, CEA, 100036, Beijing, China.

Supplementary Table 1. Atomic Populations (Mulliken) of CaC_2O_5-$I\bar{4}2d$ at 34, 45, 70, 85 and 100 GPa. Different C-O and Ca-O bonds exist in the CaC_2O_5 structure and are labelled as Ca--O^a, Ca--O^b, Ca--O^c, and C--O^a, C--O^b, C--O^c.

Pressure	Species	s	p	d	Totall	Charge(e)	Bond	Populaton	Length( )
	C	0.89	2.30	0.00	3.19	0.81	C--O^a	0.82	1.284
							C--O^b	0.58	1.400
	O^a	1.78	4.93	0.00	6.71	-0.71		0.55	1.417
							C--O^c	0.57	1.413
34 GPa	O^b	1.76	4.74	0.00	6.51	-0.51	Ca--O^a	0.08	2.255
								0.06	2.242
	O^c	1.77	4.74	0.00	6.51	-0.51		0.05	2.234
							Ca--O^b	0.06	2.498
	Ca	2.06	6.00	0.61	8.67	1.33	Ca--O^c	0.02	2.778
	C	0.89	2.31	0.00	3.20	0.80	C--O^a	0.83	1.276
							C--O^b	0.59	1.392
	O^a	1.78	4.92	0.00	6.70	-0.70		0.55	1.411
							C--O^c	0.57	1.408
45 GPa	O^b	1.76	4.74	0.00	6.50	-0.50	Ca--O^a	0.07	2.215
								0.05	2.204
	O^c	1.77	4.74	0.00	6.51	-0.51		0.05	2.200
							Ca--O^b	0.06	2.440
	Ca	2.04	6.00	0.64	8.69	1.31	Ca--O^c	0.02	2.727
	C	0.88	2.33	0.00	3.21	0.79	C--O^a	0.85	1.261
							C--O^b	0.60	1.375
	O^a	1.77	4.91	0.00	6.68	-0.68		0.56	1.398
							C--O^c	0.58	1.396
70 GPa	O^b	1.75	4.75	0.00	6.50	-0.50	Ca--O^a	0.06	2.146
								0.04	2.140
	O^c	1.76	4.75	0.00	6.51	-0.51		0.03	2.141
							Ca--O^b	0.06	2.341
	Ca	2.00	6.00	0.71	8.72	1.28	Ca--O^c	0.02	2.636
	C	0.87	2.34	0.00	3.21	0.79	C--O^a	0.86	1.256
							C--O^b	0.61	1.366
	O^a	1.76	4.90	0.00	6.67	-0.67		0.56	1.391
							C--O^c	0.58	1.389
85 GPa	O^b	1.75	4.75	0.00	6.50	-0.50	Ca--O^a	0.05	2.113
								0.03	2.109
	O^c	1.76	4.75	0.00	6.51	-0.51		0.02	2.113
							Ca--O^b	0.07	2.297
	Ca	1.99	6.00	0.75	8.73	1.27	Ca--O^c	0.02	2.592
	C	0.87	2.35	0.00	3.22	0.78	C--O^a	0.86	1.246
							C--O^b	0.61	1.359
	O^a	1.76	4.90	0.00	6.66	-0.66		0.56	1.385
							C--O^c	0.58	1.383
100 GPa	O^b	1.74	4.75	0.00	6.50	-0.50	Ca--O^a	0.04	2.085
								0.02	2.083
	O^c	1.76	4.75	0.00	6.51	-0.51		0.01	2.088
							Ca--O^b	0.07	2.262
	Ca	1.97	6.00	0.78	8.75	1.25	Ca--O^c	0.02	2.554

Supplementary Table 2. Structural parameters of CaC_2O_5-minerals studied in this work.

Space group	Pressure (GPa)	Lattice parameters a/Å	b/Å	c/Å	Angle/deg	Atom	Coordinates x	y	z
*C*2	100	6.891	3.231	9.073	α=γ=90	Ca1	0.00000	-0.48837	0.00000
					β=150.450	C1	0.26498	-0.30723	0.59898
						O1	1.50173	-0.49821	0.83667
						O2	0.02769	-0.53674	0.35043
						O3	0.50000	-0.56397	0.50000
*C*2-*l*	50	11.999	6.925	6.925	α=γ=90	Ca1	0.74997	0.37770	0.03949
					β=125.242	Ca2	0.00000	0.66723	0.50000
						Ca3	0.00000	0.58803	0.00000
						C1	-0.04139	0.29397	0.69222
						C2	0.79139	0.11130	0.37506
						C3	0.20863	0.14404	0.29231
						C4	0.54143	0.46131	0.27506
						O1	-0.03985	0.12763	0.21017
						O2	0.28989	0.12767	0.53988
						O3	0.37763	0.45309	0.47621
						O4	0.87764	0.30221	0.77905
						O5	0.12764	0.27907	0.55220
						O6	0.62769	0.47624	0.20313
						O7	0.03199	0.44508	0.75422
						O8	0.71803	0.09993	0.15058
						O9	0.28201	0.15544	0.21457
						O10	0.46806	0.31019	0.19026
Cc	15	7.425	10.415	4.541	α=γ=90	Ca1	0.25674	0.14466	-0.05315
					β=121.197	C1	0.62321	0.11812	0.75784
						C2	-0.02806	0.11398	0.23315
						O1	0.15862	0.07499	0.34546
						O2	0.55132	0.18805	0.49693
						O3	0.41853	0.32557	-0.08850
						O4	0.03845	0.42585	0.40741
						O5	0.33820	0.41133	0.39885
*Fdd*2	60	13.972	5.607	5.662	α=β=γ=90	Ca1	0.25000	0.75000	0.54067
						C1	0.16828	0.23039	0.58324
						O1	0.15643	0.96005	0.17568
						O2	0.32720	0.93499	0.23590
						O3	0.50000	0.00000	0.69348
*I*42*d*	34	7.063	7.063	10.002	α=β=γ=90	Ca1	0.46344	-0.25000	0.62500
						C1	0.83533	-0.01521	0.58257
						O1	0.82767	0.14979	0.50211
						O2	0.97466	0.3138	0.3444
						O3	1.00000	0.00000	0.66215
Pc	80	4.537	3.829	6.061	α=γ=90	Ca1	0.25562	0.29059	0.43657
					β=94.966	C1	0.84792	0.81926	0.34905
						C2	0.65986	0.68053	0.67894
						O1	0.75594	0.15956	0.38169
						O2	0.60401	0.34433	0.74339
						O3	0.41606	0.79675	0.59280
						O4	0.08746	0.79265	0.25796
						O5	0.89953	0.68943	0.55960

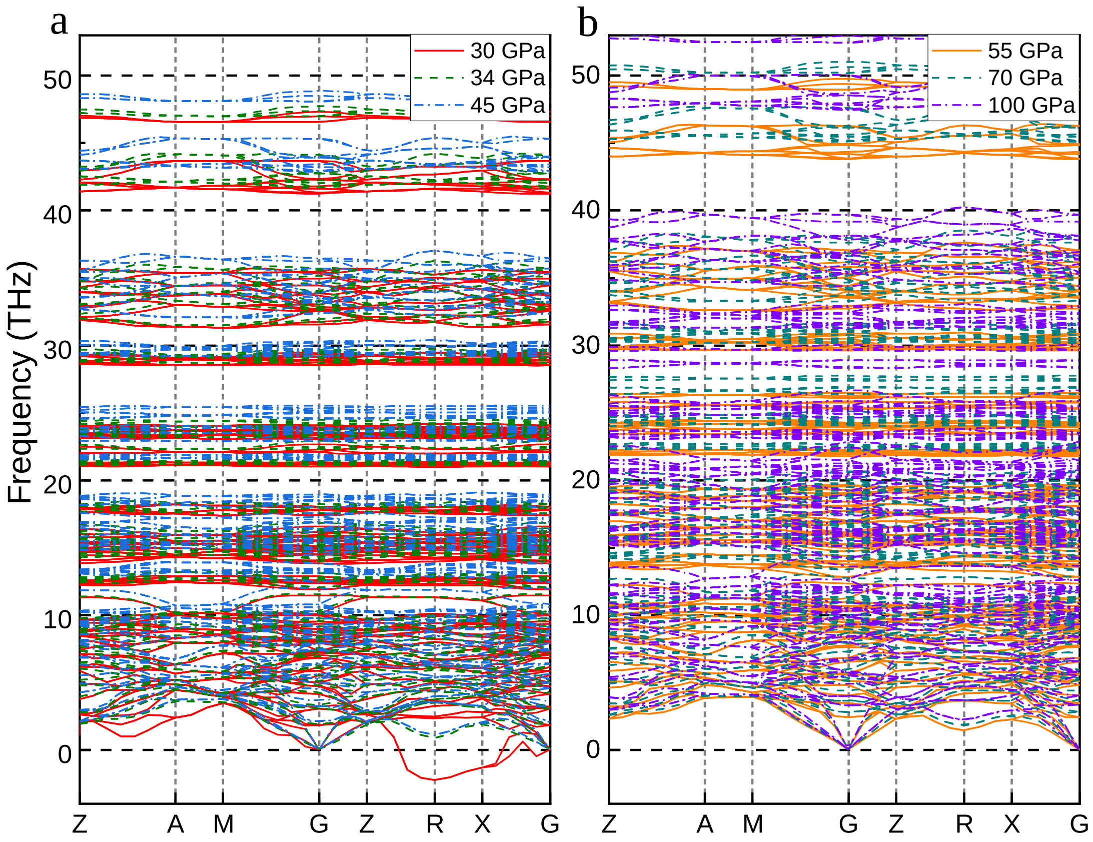

Supplementary Fig. 1 | Dynamic stability of CaC_2O_5-$\boldsymbol{I\bar{4}2d}$. a Phonon dispersion relations along select high-symmetry points in the Brillouin zone for CaC_2O_5-$I\bar{4}2d$ at 30, 34 and 45 GPa, and **b** 55, 70 and 100 GPa.

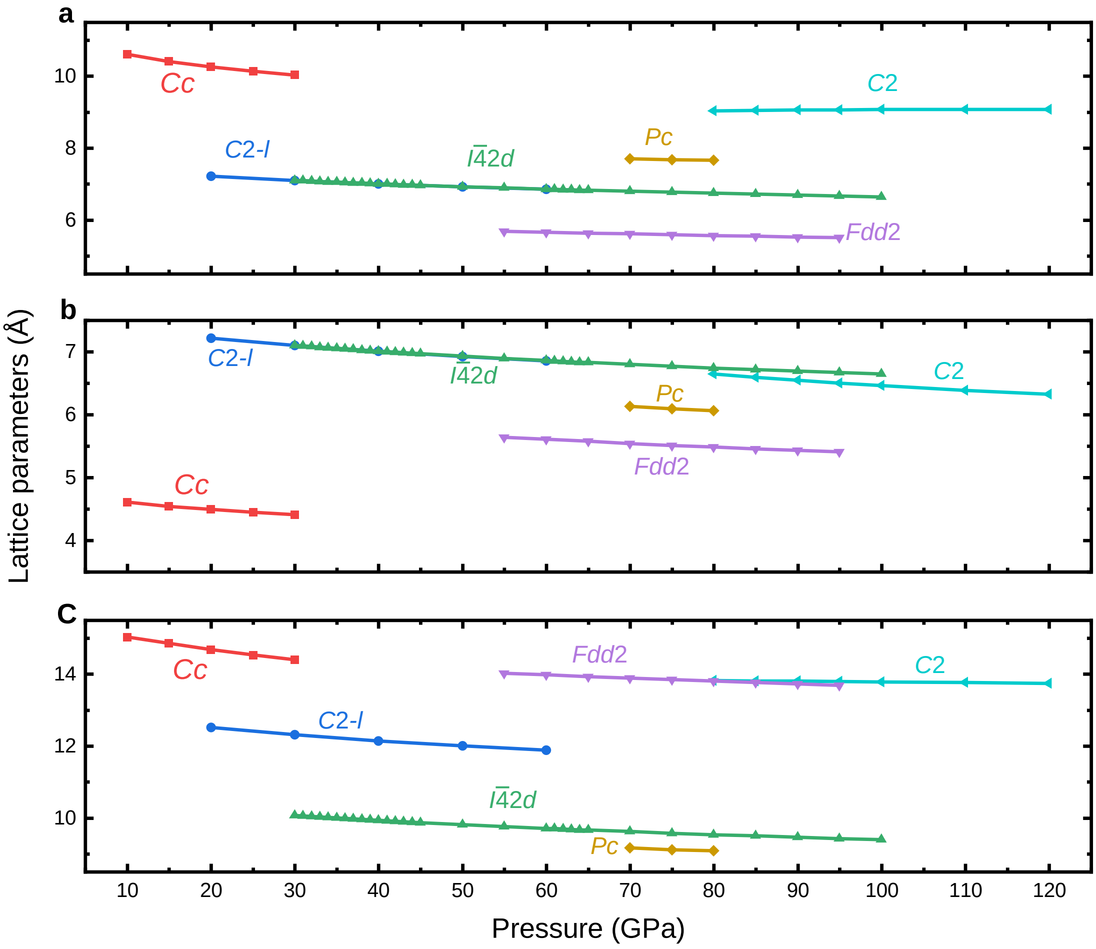

Supplementary Fig. 2 | Lattice parameters of CaC_2O_5s. a Lattice parameters *a* of CaC_2O_5s at 10 to 120 GPa. **b** Lattice parameters *b* of CaC_2O_5s at 10 to 120 GPa. **c** Lattice parameters *c* of CaC_2O_5s at 10 to 120 GPa.

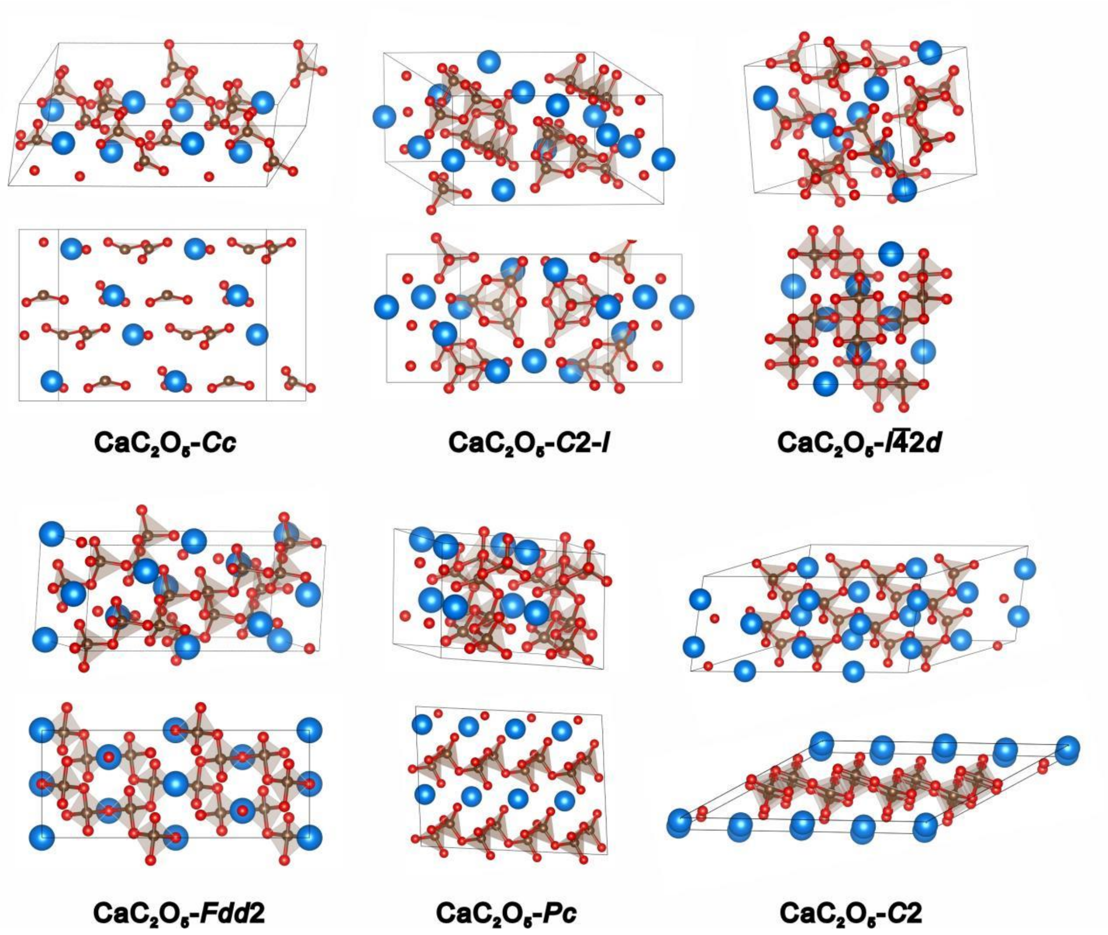

Supplementary Fig. 3 | Structural of CaC_2O_5s studied in this work. Each mineral has two different perspectives of the crystal lattice images.
